# Better health intelligence: a new era for civil registration and vital statistics?

**DOI:** 10.1186/s12916-015-0333-4

**Published:** 2015-04-07

**Authors:** Alan D Lopez, Philip W Setel

**Affiliations:** Melbourne School of Population and Global Health, University of Melbourne, 207 Bouverie Street, Carlton, 3053 Victoria Australia; The Union North America, 61 Broadway, Suite 1720, New York, NY 10006 USA

**Keywords:** Bloomberg, Causes of death, Civil registration, Vital statistics

## Abstract

The impetus and opportunities for improving birth, death, and cause of death data have never been more propitious. Renewed country commitment to strengthen vital registration systems is clearly evident, supported by nascent regional coalitions of technical and development organisations. The announcement of a major new investment by Bloomberg Philanthropies to strengthen data systems and capacity in selected countries has the potential to catalyse and realise significant improvements in the availability and quality of data for health. This will require technical leadership, strategic intervention choices, strong country partnerships, and efficient delivery and management of multiple technical interventions across participating countries.

## Background

The role that reliable, timely, and relevant information can play in improving the health of populations is unequivocal. Good, or better, health outcomes are greatly influenced by bold, informed, and effective health policies and practices, delivered by a health system that makes the most of health resources by maximizing efficiency and promoting greater equality. In order to do so, the health system, and health policy debates more broadly, rely on a functioning health information system (HIS) that generates critical data when and where needed and sufficiently disaggregated to guide policy responses. Having this information is, of course, no guarantee that it will be used, or used effectively, but not having it is a sure way to hinder health development. Indeed, very soon after the launch of the Millennium Development Goals (MDGs) in 2000 it became clear that without more effective HIS, countries could not adequately monitor progress towards these or other development goals. Not surprisingly, the World Health Organization (WHO) subsequently identified a functioning HIS as one of the six fundamental building blocks of a good health system [1].

There has been much debate about what exactly a good HIS comprises [1-3] (Box 1). Perhaps the most recent and comprehensive contribution to this discourse is the excellent *Framework and Standards for Country Health Information Systems* produced by the now defunct Health Metrics Network (HMN) in 2008. This framework identified six critical data sources for a national HIS; three (resource records, service records, individual records) derived from hospitals and other institutions, and three (censuses, civil registration, population surveys) generating data (in principle at least) on all individuals within a defined population (either total or sample). While the HMN document arguably captures the vast majority of health information subsystems that a country might need to effectively manage its health system, it offers little guidance to countries about which data sources are the highest priority for investment, and why. Various agencies, including the HMN, the WHO, and other development partners, have at different times emphasized various information system components as a priority, often connected to global development goals or major disease control strategies [2,3].

More recent efforts have attempted to identify what information is essential to guide health policy debates and health services delivery. One outcome has been the promotion of a HIS strategy focused on monitoring the effective coverage of key health interventions against the major causes of disease burden in the population [4-6]. While there is some logic in this approach, it is not without measurement challenges. Reliably estimating the burden of (fatal and non-fatal) illness and injury in a population requires prioritization of several data collections, and switching from government reported data on intervention coverage to accurately measuring true, or effective coverage in the population, will most likely require a significant survey program. Thus, while appealing, is this the optimum and most cost-effective HIS strategy to guide health and development efforts?

As strategies for HIS support for health policies have been evolving, governments and the international development community alike have become more and more aware that knowing the rate at which people are dying, at what ages, from what causes, and how rapidly this epidemiological situation is changing, has become a central, unavoidable priority for health policy debates in all countries. This has undoubtedly been influenced by a strong interest in knowing how well we have progressed with child survival strategies over the past few decades, but also to inform the massive investments that have gone into controlling HIV/AIDS in particular. So, if a functioning vital registration system that captures and correctly certifies the cause of all deaths might be considered as the cornerstone of any HIS, what progress has been made? What remains to be done? And how can we accelerate the process of system improvement? This commentary will discuss these issues in the context of the Bloomberg Data for Health Initiative.

### Limited progress with civil registration and vital statistics systems

Despite their obvious utility for guiding health policy decisions, informing health research priorities, and for monitoring progress with specific disease and injury control strategies, too few low- and middle-income countries have functioning vital registration systems that are fit for purpose. In 2007, Mahapatra et al. [7] lamented the slow rate of progress with civil registration and vital statistics (CRVS) systems over the period since 1980, and a recent analysis has suggested that there has been little progress since then [8]. Only about one in three deaths worldwide are registered by death notification systems today, and in some cases up to half of these deaths are attributed to one or more ‘garbage’ codes that are utterly uninformative for public policy [9]. This is not to say that there has not been good progress in strengthening vital registration systems in some countries. However, these are the exception and in some cases the performance of the vital registration system has markedly declined or it has even ceased producing data altogether [8,9].

The reasons for such indifferent progress with CRVS systems are undoubtedly multifaceted. Certainly, the complexity of a HIS, and the absence of a rational and compelling basis for choosing among the plethora of HIS investments, might have driven inaction. It is clear that countries cannot change decades, if not centuries, of HIS inertia overnight. Further, the focused, strategic, and practical guidance that countries needed was not emerging from the international health statistical architecture. Nevertheless, one might have expected that the repeated references to the role of good cause of death data in combatting several contemporary global health challenges would have led to an acceleration of efforts [10,11]. A good example is the WHO’s global non-communicable disease (NCD) control strategy adopted by the United Nations General Assembly in 2011, a key aspect of which is monitoring premature mortality from major NCDs [12]. Few would doubt that preventing premature death from cancer, major vascular disease, diabetes, liver disease, chronic kidney disease, or chronic obstructive lung disease and related conditions is likely to be a dominant concern for most low- and middle-income countries over the next few decades. Despite this, we remain remarkably ignorant about mortality trends from NCDs in precisely those countries where they are most likely to accelerate [13]. That ignorance can only be addressed by rapidly developing country capacity for routinely and accurately registering and certifying the cause of all deaths within a functioning vital registration system. No other data collection vehicle can generate this essential health intelligence on a continuous basis and in a comparable and disaggregated fashion for the entire population [14].

### Strengthening CRVS systems**:** time for new solutions?

The task ahead is enormous. The WHO has effectively led the development of successive revisions to the International Classification of Diseases. Unfortunately, it has not had the resources to ensure that the public health application of the International Classification of Diseases in countries yields the data essential for public policy support. It is also not always clear which agency – WHO, the United Nations Statistics Division, or UNICEF – has responsibility for strengthening which aspects of death registration systems, and perhaps it does not matter. What is clear is that the current rate of slow progress will not meet the needs of countries soon enough, or adequately enough, to help them build the evidence base they need to accelerate health development and address the looming, if not actual, epidemiological challenges they are facing.

A new strategy, with new partners, is required. To some extent this is already occurring. Several regions have established effective regional coalitions led by the United Nations Economic Commissions (Africa, Asia-Pacific) or by a WHO Regional Office (Eastern Mediterranean) that have enlisted ministerial support to strengthen CRVS systems, following the strategic directions and pathways identified in the set of tools that have recently become available [15,16]. Yet, even these promising and purposeful initiatives risk stalling unless greater resources, and especially technical support and leadership, can be efficiently identified and allocated. The recent initiative led by the World Bank to develop the investment case for CRVS systems is an important step in this direction [17], as is the recent announcement by the Canadian Department of Foreign Affairs, Trade, and Development to explicitly support Canadian institutions to develop CRVS systems skills and knowledge in countries that most need them [18].

### The Bloomberg Data for Health initiative

A new initiative, recently announced by Bloomberg Philanthropies in New York, in collaboration with the Australian Department of Foreign Affairs and Trade, establishes a global partnership that will bring together key expertise in CRVS system development to rapidly strengthen vital registration systems [19]. Indeed, the partnership between Bloomberg and the Australian Department of Foreign Affairs and Trade is itself a welcome innovation, joining global philanthropy with an established history of NCD control investments, with a major bilateral government development partner. This is certainly novel, and the broader scope of the Bloomberg Data for Health Initiative, involving intensified support also for NCD surveillance and, importantly, in the use of data to guide policy, is absolutely correct and welcome.

However, novelty alone will not ensure success. A priority will be to strategically engage, support, and complement other development partners active in countries to more effectively respond to country needs for basic health information. Without this critical health intelligence, governments and donors are ‘flying blind’ about where to target limited public health resources.

The Initiative is predicated on the belief that better public health information would facilitate more informed health policy and health system responses, increasing the likelihood of better population health outcomes. It represents an extraordinary opportunity to increase the availability, quality, use, and country-level ownership of critical public health data to improve decision-making, track disease and injury trends, and plan interventions. The Initiative will bring technical leadership and resources to help countries better prioritize data for health challenges in countries. Importantly, it can demonstrate that success in strengthening CRVS systems can be accomplished in a short space of time, and ensure that the skills, knowledge, and practices left behind will be sustainable, appreciated, and used for country health development.

## Conclusions

After decades of stagnation, countries, regional organizations, bilateral donors, major global development partners, and philanthropy are now coalescing and aligning efforts to address what has been labelled as the “*single most critical failure of development over the past 30 years*”, namely improving birth, death, and cause of death data systems [20]. Countries, and the global development community more broadly, have been deciding and implementing policy often on the basis of very poor evidence, or no evidence at all. That is hardly good development policy, and ought to be completely unacceptable in this era of data revolution. Nor does it facilitate serious and responsive monitoring of progress towards key development goals such as the Millennium Development Goals or the Sustainable Development Goals under consideration.

The announcement of a significant investment from Bloomberg Philanthropies, among others, to improve data for health is thus not only welcome, but timely. There are reasons for optimism that such bold and innovative investments in strengthening data systems and the use of data for public policy could lead to substantially better progress with CRVS systems than what has been observed over the past few decades. This is a new partnership and it should do business differently, focusing on harnessing the best available global technical expertise and leadership to inspire countries, support them with the implementation of targeted CRVS interventions, and build CRVS system capacity in a sustainable manner. That is now possible, possibly for the first time ever; we all have a collective responsibility to better prepare countries in the generation and use of data for health development.

## Box 1: Health Information Systems

A Health Information System (HIS) is an integrated system of data sources and other knowledge that produces the information and skills required by countries to effectively manage their health system and to guide the formulation and evaluation of health policies.

A good HIS is one that does so efficiently, reliably, continuously, and comprehensively, producing statistics and knowledge that is directly relevant to protecting and promoting the population’s health. In particular, data need to be timely, disaggregated, accurate, and relevant to have maximum policy benefit.

Up-to-date, reliable information on who is dying of what in a population, and at what age, is arguably the cornerstone of any HIS since this intelligence is fundamentally important for policies to prevent premature death. In most low- and middle-income countries, cause of death data systems are too weak to provide this information with the result that health policies and planning are often based on very limited evidence.

## Abbreviations

CRVS: Civil registration and vital statistics (vital registration)
HIS: Health Information System
HMN: Health Metrics Network
NCD: Non-communicable disease
WHO: World Health Organization

## References

[1] World Health Organisation (2007). Everybody’s business: strengthening health systems to improve health outcomes: WHO’s framework for action.

[2] Health Metrics Network. Framework and standards for country health information systems. Second ed. Geneva: World Health Organisation; 2008.

[3] Health Metrics Network (2011). HMN Corporate Plan, 2012–2013.

[4] Murray CJL (2009). Assessing health systems performance using information on effective coverage of interventions. Working paper series No.3.

[5] Lozano R, Soliz P, Gakidou E, Abbott-Klafter J, Feehan D, Vidal C (2006). Benchmarking of performance of Mexican states with effective coverage. Lancet..

[6] Byass P, de Savigny D, Lopez AD (2014). Essential evidence for guiding health system priorities and policies: anticipating epidemiological transition in Africa. Global Health Action..

[7] Mahapatra P, Shibuya K, Lopez AD, Coullare F, Notzon FC, Rao C (2007). Civil registration systems and vital statistics: successes and missed opportunities. Lancet..

[8] Mikkelsen L, Philips DE, Abouzahr C, Setel PW, de Savigny D, Lozano R, et al. Monitoring data quality and progress with civil registration and vital statistics systems: a global assessment. Lancet. In press. 10.1016/S0140-6736(15)60171-4 25971218

[9] Philips DE, Lozano R, Naghavi M, Atkinson C, Gonzales-Medina D, Mikkelsen L (2014). A composite metric for assessing data on mortality and causes of death: the Vital Statistics Performance Index. Pop Hlth Metr..

[10] World Health Organization. Accountability for women’s and children’s health. Independent expert review group on women’s and children’s’ health. http://www.who.int/woman_child_accountability/ierg/en/.

[11] United Nations. The Millennium Development Goals report 2013. New York: United Nations, 2013. http://www.un.org/millenniumgoals/pdf/report-2013/mdg-report-2013-english.pdf. Accessed March 15, 2015.

[12] Nations U (2011). Outcome document of the high-level meeting of the General Assembly on the comprehensive review and assessment of progress achieved in the prevention and control of non-communicable diseases.

[13] Naghavi M, Wang H, Lozano R, Davis A, Liang X, Zhou M (2015). Global, regional, and national age-sex specific all-cause and cause-specific mortality for 240 causes of death, 1990–2013: a systematic analysis for the Global Burden of Disease Study 2013. Lancet..

[14] Hill K, Lopez AD, Shibuya K, Jha P (2007). Interim measures for meeting needs for health sector data: births, deaths, and causes of death. Lancet..

[15] Mikkelsen L, Lopez AD (2010). Improving the quality of birth, death and cause-of-death information: guidance for a standards-based review of country practices. Working Paper series No. 1.

[16] Abouzahr C, Lopez AD, Mikkelsen L, Rampatige R, Schmider A, Upham S (2012). Strengthening practice and systems in civil registration and vital statistics: a resource kit. Working Paper series No. 19.

[17] World Bank and World Health Organisation. Global civil registration and vital statistics: Scaling-up investment plan 2014–2025. http://www.who.int/healthinfo/civil_registration/crvs_meeting_apr2014_presentation_session3.zip?ua=1.

[18] Canadian Department of Foreign Affairs, Trade and Development. Partnerships for strengthening maternal, newborn and child health: call for proposals. www.international.gc.ca/development-developpement/partners-partenaires/calls-appels/psmnch-prsmne.aspx?lang=eng. Accessed March 15, 2015.

[19] Bloomberg Philanthropies. Bloomberg Philanthropies Launches $100 Million Data for Health Program in Developing Countries. March 23, 2015. http://www.bloomberg.org/press/releases/bloomberg-philanthropies-launches-100-million-data-health-program-developing-countries/.

[20] Setel PW, Macfarlane SB, Szreter S, Mikkelsen L, Jha P, Stout S (2007). A scandal of invisibility: making everyone count by counting everyone. Lancet..

